# Partial nephrectomy for T3aN0M0 renal cell carcinoma: shall we step forward?

**DOI:** 10.1590/S1677-5538.IBJU.2016.0598

**Published:** 2017

**Authors:** Ding Peng, Zhi-song He, Xue-song Li, Qi Tang, Lei Zhang, Kai-wei Yang, Xiao-teng Yu, Cui-jian Zhang, Li-qun Zhou

**Affiliations:** 1 Department of Urology, Institute of Urology, Peking University First Hospital, Beijing, China

**Keywords:** Carcinoma, Renal Cell, Nephrectomy, Patients

## Abstract

**Objectives:**

To evaluate the prognosis of non-metastatic T3a renal cell carcinoma (RCC) with partial nephrectomy (PN).

**Patients and Methods:**

We retrospectively evaluated 125 patients with non-metastatic T3a RCC. Patients undergoing PN and radical nephrectomy (RN) were strictly matched by clinic-pathologic characteristics. Log-rank test and Cox regression model were used for univariate and multivariate analysis.

**Results:**

18 pair patients were matched and the median follow-up was 35.5 (10-86) months. PN patients had a higher postoperative eGFR than RN patients (P=0.034). Cancer-specific survival (CSS) and recurrence-free survival (RFS) did not differ between two groups (P=0.305 and P=0.524). On multivariate analysis, CSS decreased with positive surgical margin and anemia (both P <0.01) and RFS decreased with Furhman grade, positive surgical margin, and anemia (all P<0.01).

**Conclusions:**

For patients with non-metastatic pT3a RCC, PN may be a possible option for similar oncology outcomes and better renal function.

## INTRODUCTION

Radical nephrectomy (RN) used to be the standard therapeutic option for localized and locally advanced renal cell carcinoma (RCC) (1). In recent years, the oncology outcomes with partial nephrectomy (PN) were found not worse than those of RN (2). Moreover, PN may improve overall survival by preventing cardiovascular events caused by chronic kidney disease (CKD) for that the incidence of CKD was lower with PN than RN (3-5).

PN was recommended by the European Association of Urology and National Comprehensive Cancer Network guidelines as the preferred option for tumor type 1a-b (T1a-b) RCC 2 (1, 6). In recent years, several studies expanded the application further to T2 RCC patients (7). However, whether PN is a possible option for non-metastatic T3a RCC is unknown. Actually, some non-metastatic patients with pT3a RCC (perinephric and renal sinus fat invasion) have undergone PN for various reasons.

The aim of this study was to analyze the prognostic differences in T3a RCC patients who underwent PN or RN with a strictly case-matched design.

## PATIENTS AND METHODS

We searched the renal cancer database in Peking University First Hospital for cases occurring from 2007 to 2012 and retrospectively identified 2651 RCC patients who underwent nephrectomy, including PN and RN. The study received institutional review board approval. According to the 2010 American Joint Committee on Cancer (AJCC) TNM staging, the patients included 125 with non-metastatic pathological T3a (pT3a) RCC; 18 underwent PN. Patients who underwent PN and RN were exactly matched by gender, age, tumor size, American Society of Anesthesiologists (ASA) score, pathological subtype, surgical margin status, tumor invasion status and Fuhrman grade (Table-1). When more than one RN patient with criteria identified was matched, we chose patients with a smaller difference in tumor size with PN patients.

**Table 1:** Criteria for pair matching.

Criterion	Difference allowed
Age	<10 years
Gender	Identical
Tumor size	<3 cm
ASA score	Identical
Pathological subtype	Identical
Tumor invasion status	Identical
Surgical margin status	Identical
Fuhrman grade	Identical

Complete preoperative examinations included chest X-ray, abdominal ultrasonography, abdominal CT, laboratory examinations and other necessary exams for preoperative evaluation. Pathological specimens were assessed by at least two experienced pathologists to confirm the pathologic subtype, surgical margin status, tumor invasion status and lymph-node metastasis status. Histological subtype and Fuhrman grade were stratified according to the 2004 WHO classification system and 1997 WHO recommended standards, respectively. According to 2010 AJCC TNM staging system, T3a staging was diagnosed when the tumor grossly extended into the renal vein or its segmented (muscle-containing) branches or invaded the perirenal and/or renal sinus fat but not beyond Gerota's fascia.

Patients were followed up by the standard strategy for outpatients in our institution, every 3 months post-operatively for the first 2 years and every 6 months for the next 3 years. From the fifth year and thereafter, patients were followed up annually. The general follow-up included imaging examinations (chest X-ray, abdominal ultrasonography or CT) and laboratory examinations (blood, urine and biochemistry). The outcomes investigated during follow-up included cancer-specific survival (CSS) and recurrence-free survival (RFS). The period from the surgery date to the date of recurrence, death or last follow-up was calculated as the follow-up time.

Statistics analysis involved use of SPSS 22.0 (SPSS Inc., Chicago, IL). Student t test was used to compare continuous variables and chi-square test to compare categorical variables. Survival was estimated by Kaplan-Meier method and log-rank test was used for survival difference analysis and univariate analysis. Variables with significant differences on univariate analysis for all T3aN0M0 patients were included in Cox multivariate regression analysis. All comparisons involved two-tailed tests and P<0.05 was considered statistically significant.

## RESULTS

A total of 18 patients with non-metastatic pT_3a_ renal cell carcinoma underwent PN in our institution. The reasons were solitary kidney (n=3), renal insufficiency (n=3) and preoperative diagnosis of clinical T1 or T2 (cT1 or cT2). From the matching variables, 18 patients who underwent RN were chosen as the control group. For the 36 patients, the median age was 68.5 years (range 36-85); 28 (77.8%) were male and median follow-up was 35.5 months (10-86). The two groups did not differ in baseline characteristics (P >0.05) or some post-operative features such as blood loss (P=0.845), operative time (P=0.110), drainage-tube indwelling time (P=0.778) and post-operative stay (P=0.540). The preoperative eGFR in both groups is not significant different (P=0.357) and the postoperative eGFR in PN group is higher than RN group (P=0.034) (Table-2).

**Table 2:** Baseline characteristics and postoperative outcomes for patients with non-metastatic pT3a renal cell carcinoma (RCC) who underwent radical nephrectomy (RN) and partial nephrectomy (PN) (n=18 each).

Variable		RN	PN	P
Age		64.94±12.62	60.89±13.99	0.368
Male gender		14	14	**Matched**
**Histopathologic subtype**				**Matched**
	ccRCC	13	13	
	non-ccRCC	5	5	
**Fuhrman grade**				**Matched**
	1	1	1	
	2	11	11	
	3	6	6	
**ASA score**				**Matched**
	1	1	1	
	2	13	13	
	3	4	4	
Tumor size		5.03±1.42	5.27±1.50	0.644
**Tumor invasion**				**Matched**
	Fat	17	17	
	Renal vein	1	1	
**Surgical margin**				**Matched**
	Positive	1	1	
	Negative	17	17	
Blood loss (mL)		248.89±570.22	287.22±598.03	0.845
Operative time (min)		157.44±45.04	131.61±49.39	0.110
Indwelling drainage tube time (day)		4.94±4.14	4.61±2.77	0.778
Postoperative stay (days)		7.61±2.50	6.44±3.41	0.250
Preoperative eGFR (mL/min/1.73m^2^)		82.60±26.53	73.99±27.97	0.357
Postoperative eGFR (mL/min/1.73m^2^)		52.35±17.21	68.38±24.40	**0.034** *

Data are mean±SD or number.

* *P*<0.05

**ccRCC =** clear cell renal cell carcinoma; **RN =** radical nephrectomy; **PN =** partial nephrectomy; **ASA =** American Society of Anesthesiologists

**Figure 1:**
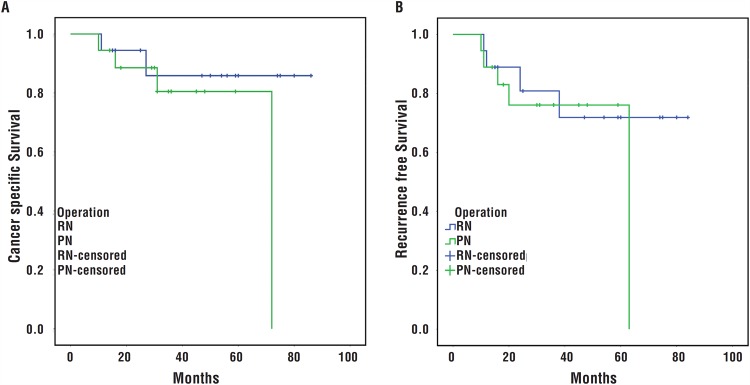
Kaplan-Meier survival analysis of patients with non-metastatic pT3a renal cell carcinoma (RCC) who underwent radical nephrectomy (RN) and partial nephrectomy (PN). A) Cancer-specific survival (P=0.305); B) Recurrence-free survival (P=0.524).

At the end of follow-up, 4 (22.2%) and 2 (11.1%) patients in the PN and RN groups died due to the disease and 5 (27.8%) and 4 (22.2%) showed recurrence, respectively. The patients who died in the PN group were 1 with renal insufficiency, 3 up-graded after operation and the patients with recurrence in PN group were 1 for renal insufficiency, 1 with solitary kidney and 3 up-graded after operation. The estimated 5-year CSS for the PN and RN patients was 80.5% and 85.9%, and the estimated 5-year RFS was 76% and 80.8%. CSS (P=0.305) and RFS (P=0.524) did not differ between the two groups on log-rank testing (Figure-1).

Univariate analysis revealed that tumor invasion status, Fuhrman grade, positive surgical margin, hypoalbuminaemia and anemia were significantly associated with CSS and RFS for all T3aN0M0 patients (n=125) (Table-3). Surgical type (RN vs. PN) was not significantly associated with CSS and RFS. On Cox multivariate regression analysis, CSS was decreased with positive surgical margin (P=0.000) and anemia (P=0.003) and RFS was decreased with Fuhrman grade (P=0.001), positive surgical margin (P=0.003) and anemia (P=0.004) (Table-4).

**Table 3:** Univariate analysis of clinicopathological variables associated with cancer-specific survival (CSS) and recurrence-free survival (RFS) at 5 years for all T_3a_N_0_M_0_ patients (n=125).

Variable		n		CSS			RFS	
			CSS	χ^2^	*P*	*RFS*	χ^2^	P
**Gender**				2.067	0.151		2.840	0.092
	Male	87	65.4			53.1		
	Female	38	74.2			69.7		
**Age, years**				0.736	0.391		0.400	0.527
	<55	49	69.6			59.1		
	≥55	76	67.5			57.7		
**BMI**				0.146	0.703		0.230	0.632
	<25	68	64.3			59.8		
	≥25	57	72.8			55.9		
**Tumor invasion**				9.772	**0.002** *		4.231	**0.040** *
	Fat	89	78.6			64.6		
	Renal vein	36	46.3			42.9		
	Histopathologic			0.267	0.605		0.801	0.371
	subtype							
	ccRCC	111	68.3			56.0		
	non-ccRCC	14	68.8			77.4		
	Location			1.206	0.272		0.905	0.341
	Left	66	62.9			54.9		
	Right	59	74.4			62.9		
**Tumor size, cm**				0.007	0.935		0.310	0.578
	≤4	9	83.3			58.3		
	>4	116	68.9			57.8		
**Fuhrman grade**				8.968	**0.003** *		16.623	**<0.001** *
	1&2	60	85.7			79.6		
	3&4	65	53.9			39.5		
**ASA score**				0.000	0.993		0.834	0.361
	1&2	113	67.4			58.6		
	3&4	12	76.2			60.0		
**Surgical type**				0.021	0.885		0.101	0.751
	RN	107	67.3			56.6		
	PN	18	80.5			76		
**Surgical margin**				46.172	**<0.001** *		16.169	**<0.001** *
	Positive	2	0			0		
	Negative	123	69.1			59.2		
**Surgical approach**				0.061	0.805		2.023	0.155
	Open	80	67.2			54.5		
	Laparoscopic	45	74			67.8		
**Hypoalbuminaemia**				10.934	**0.001** *		4.394	**0.036** *
	No	115	71.9			60.2		
	Yes	10	20			28.6		
**Anemia**				22.03	**<0.001** *		13.349	**<0.001** *
	No	102	75.6			63.7		
	Yes	23	33			29.8		

* P<0.05

**BMI =** body mass index; ccRCC = clear cell renal cell carcinoma; **RN =** radical nephrectomy; **PN =** partial nephrectomy; **ASA =** American Society of Anesthesiologists

**Table 4:** Multivariate analysis of clinicopathological variables for CSS and RFS for all T_3a_N_0_M_0_ patients (n=125).

Variable			CSS			RFS	
		*HR*	*(95% CI)*	*P*	*HR*	*(95% CI)*	*P*
**Tumor invasion**							
	Fat	1			1		
	Renal vein	1.386	0.580-3.315	0.463	1.201	0.593-2.432	0.610
**Fuhrman grade**							
	1&2	1			1		
	3&4	1.865	0.701-4.961	0.212	3.688	1.651-8.239	**0.001** *
**Surgical margin**							
	Positive	41.318	6.686-255.331	**<0.001** *	10.861	2.231-52.873	**0.003** *
	Negative	1			1		
**Serum album, g/L**							
	≥35	1			1		
	<35	1.678	0.580-4.855	0.339	1.300	0.431-3.922	0.642
**Anemia**							
	No	1			1		
	Yes	3.633	1.546-8.537	**0.003** *	3.201	1.464-7.000	**0.004** *

* P<0.05

**HR** = hazard ratio; **CI** = confidence interval

## DISCUSSION

From our institution's data on 125 patients with non-metastatic pT3a RCC, 18 patients with PN and 18 with RN were matched. We compared the outcomes of patients with localized RCC undergoing PN and other patients undergoing routine RN treatment to evaluate the outcomes and effectiveness of PN for non-metastatic T3a RCC. The postoperative eGFR in PN group is higher than RN group. CSS and RFS did not differ between the groups. In the multivariate analysis, positive surgical margin and anemia were independent risk factors for CSS and high Furhman grade, positive surgical margin, and anemia were risk factors for RSS.

A number of studies have shown no significant differences between PN and RN in survival with localized RCC (2, 8, 9). Moreover, several studies suggested that PN could reduce the incidence of CKD and prevent the associated cardiovascular events and improve survival quality (2, 10). However, the therapeutic recommendation is still RN for T3aN0M0 RCC (1, 6). Nevertheless, some T3a RCC patients undergo PN for various reasons such as solitary kidney and renal insufficiency. As well, a few patients were preoperatively diagnosed with cT1-2 cancer and treated with PN, which was pathologically upstaged to pT3a cancer.

In a study by Lee et al. (11), 43 (3.2%) of 1367 patients with small RCC (≤4cm) had pT3a lesions. Gorin et al. (12) retrospectively analyzed 1.096 cT1 patients after PN and found 41 (4.8%) tumors upstaged to pT3a; the 2-year RFS with pT3a tumors was 91.8%, which was lower than with pT1-2 tumors (99.2%, P=0.003). Tumor upstaging was associated with a high R.E.N.A.L (radius, exophytic/endophytic, nearness to collecting system or sinus, anterior/posterior and location relative to polar lines) nephrometry score (hazard ratio [HR] 2.97, 95% CI 1.20-7.35, P=0.02), increased tumor diameter (1.66, 1.32-2.08, P<0.001), and hilar location (2.83, 1.43-5.61, P=0.003). In another multi-institutional study, Nayak et al. (13) reported 134 (9%) of 1.448 cT1 patients with upstaging to pT3a. The 3-year RFS with upstaging was 76% as compared with 93% without upstaging (P<0.001). Disease recurrence, increasing age, Fuhrman grade and tumor size were independently associated with pathological upstaging. In our study, we found 11 of 125 (8.8%) patients with non-metastatic pT3a RCC with cT1 cancer treated by PN.

Various risk factors associated with the oncology outcomes of non-metastatic RCC include age, gender, TNM, Fuhrman grade, tumor size, histopathologic subtype, ASA score and tumor invasion status (14, 15). Therefore, strict matching should be conducted to mitigate potential selection bias caused by these features. Lee et al. (11) found no significant differences between T1a and T3a patients in overall survival (P=0.521), CSS (P=0.651) and RFS (P=0.250). However, several variables such as age (P=0.015), tumor size (P <0.001), subtype (P=0.020), and Fuhrman grade (P=0.021) differed between the 2 cohorts. Jeldres et al. (16) matched pT3a RCC by age, gender, tumor size, Fuhrman grade and histopathologic subtype to create a cohort of PN patients (n=30) and RN patients (n=63) and demonstrated no significant difference between the groups in CSS (P=0.9). The authors also included all unmatched 72PN patients and 789RN patients in the multivariate analysis and found PN not associated with worse CSS as compared with RN (HR=0.62, P=0.11). In our study, the matching criteria (gender, age, tumor size, ASA score, pathological subtype, surgical margin status, tumor invasion status and Fuhrman grade) were more stringent than that used by Jeldres et al., and we found no significant differences between the PN and RN groups in CSS (P=0.305) and RFS (P=0.524). As well, on univariate analysis of all T3aN0M0 RCC patients, surgical type (PN or RN) was not associated with CSS or RFS.

Renal insufficiency has an independent and graded association with risk of death and cardiovascular events (17, 18). RCC patients have about a 25% rate of CKD, which can lead to a series of metabolic disorders and cardiovascular events (19). PN was suggested to benefit renal function as compared with RN (20, 21). Sun et al. found a lower rate of postoperative renal events with PN versus RN (22). Yokoyama et al. (23) found RN as an independent risk factor for new-onset CKD. Jong Jin et al. (24) compared 45 T3a RCC patients who underwent PN and 298 patients who underwent RN and found that renal function, measured by postoperative creatinine (Cr) and estimated glomerular filtration rate (eGFR), was better with PN than RN (Cr: 1.07 vs. 1.37mg/dL, P=0.001; eGFR: 75.4 vs. 59.8mL/min, P <0.001). However, RFS was lower for RN than PN patients (P <0.001). Because the mean tumor size was smaller for PN than RN patients (P <0.001), the authors also analyzed RFS for patients with tumor size ≤4cm (30PN patients, 33RN patients) and found no significant difference (P=0.306). Similar to the results of patients with tumor size ≤4cm in this previous study, we found similar CSS and RFS but higher postoperative eGFR for PN than RN patients (P=0.034) and this could imply that PN may protect renal function.

Positive surgical margin was previously found significantly associated with tumor recurrence, although the development of metastases and CSS were comparable with positive and negative surgical margins (25-27). Borghesi et al. (28) reported that the overall incidence of recurrence after negative surgical margins ranged from 0% to 7%. In our study, 2 of 125 patients (1.6%) with T3aN0M0 RCC showed positive surgical margins and the variable was associated with CSS (P=0.000) and RFS (P=0.000) on Cox multiple regression analysis. However, because of the low rate of positive surgical margins in single-center T3aN0M0 RCC patients, the association with oncological outcomes demands larger cohort studies.

This study has several strengths. The experienced surgeons in a single center ensured that every patient received similar and standard treatment. Furthermore, the sequential and uniform follow-up provided high-quality data for analysis. Moreover, the characteristics of patients in the PN and RN groups were comparable with the strict case matching.

However, this study still presents several limitations. First, it was retrospective and cannot avoid the inevitable disadvantages of a retrospective study. Second, this is a small sample and single-center study, for a low ratio of T3a RCC patients undergoing PN and this cannot avoid the type 1 or 2 error. Third, the median follow-up time was short. Prospective, large-sample and multi-institutional studies are required to further test the use of PN and discover risk factors for non-metastatic T3a RCC patients.

In conclusion, this case-matched analysis demonstrates that for non-metastatic T3a RCC patients, PN may be is a possible option for similar oncology outcomes and better renal function.
